# The Oxidative Stress-Induced Increase in the Membrane Expression of the Water-Permeable Channel Aquaporin-4 in Astrocytes Is Regulated by Caveolin-1 Phosphorylation

**DOI:** 10.3389/fncel.2017.00412

**Published:** 2017-12-20

**Authors:** Chongshan Bi, Daniel K. L. Tham, Caroline Perronnet, Bharat Joshi, Ivan R. Nabi, Hakima Moukhles

**Affiliations:** Department of Cellular and Physiological Sciences, University of British Columbia, Vancouver, BC, Canada

**Keywords:** reactive oxygen species, oxidative stress, aquaporin 4, caveolin-1, astrocytes

## Abstract

The reperfusion of ischemic brain tissue following a cerebral stroke causes oxidative stress, and leads to the generation of reactive oxygen species (ROS). Apart from inflicting oxidative damage, the latter may also trigger the upregulation of aquaporin 4 (AQP4), a water-permeable channel expressed by astroglial cells of the blood-brain barrier (BBB), and contribute to edema formation, the severity of which is known to be the primary determinant of mortality and morbidity. The mechanism through which this occurs remains unknown. In the present study, we have attempted to address this question using primary astrocyte cultures treated with hydrogen peroxide (H_2_O_2_) as a model system. First, we showed that H_2_O_2_ induces a significant increase in AQP4 protein levels and that this is inhibited by the antioxidant N-acetylcysteine (NAC). Second, we demonstrated using cell surface biotinylation that H_2_O_2_ increases AQP4 cell-surface expression independently of it’s increased synthesis. In parallel, we found that caveolin-1 (Cav1) is phosphorylated in response to H_2_O_2_ and that this is reversed by the Src kinase inhibitor 4-Amino-5-(4-chlorophenyl)-7-(t-butyl)pyrazolo[3,4-d]pyrimidine (PP2). PP2 also abrogated the H_2_O_2_-induced increase in AQP4 surface levels, suggesting that  the phosphorylation of tyrosine-14 of Cav1 regulates  this  process. We  further  showed  that dominant-negative Y14F and phosphomimetic Y14D mutants caused a decrease and increase in AQP4 membrane expression respectively, and that the knockdown of Cav1 inhibits the increase in AQP4 cell-surface, expression following H_2_O_2_ treatment. Together, these findings suggest that oxidative stress-induced Cav1 phosphorylation modulates AQP4 subcellular distribution and therefore may indirectly regulate AQP4-mediated water transport.

## Introduction

Aquaporin 4 (AQP4) is the most abundant water-permeable channel found in the mammalian brain, where it is expressed within the astrocytes that form part of the blood-brain barrier (BBB) and is detected in especially high quantities at the astrocytic endfeet that encapsulate the blood vessels (Nielsen et al., 1997). It is currently uncertain whether AQP4 is also found in the endothelial cells that comprise the microvasculature: while minor quantities of the channel have been detected in adluminal sites by immunogold labeling (Amiry-Moghaddam et al., 2004), its expression here has not been verified by immunofluorescence techniques (Haj-Yasein et al., 2012). It has been determined that the ablation of AQP4 reduces the water permeability of the astrocytic plasma membrane by as much as seven times (Solenov et al., 2004). Given its localization and its properties, it is thus currently believed that AQP4 contributes to the maintenance of osmostasis in the brain by mediating the bi-directional exchange of water with the the bloodstream (Jung et al., 1994). The AQP4 knock out mouse exhibits numerous olfactory and auditory defects, underscoring its importance (Lu et al., 1996, 2008).

In the aftermath of an ischemic stroke, the infarct region and the areas that surround it undergo a period of edematous swelling. The initial phase of this swelling, termed “cytotoxic edema” develops when the anoxic conditions brought on by the cessation of blood flow cause astrocytes to enter a state of metabolic derangement. This results in the influx and accumulation of extracellular Na^+^, followed soon after by Cl^−^ and water, driven by the electrochemical gradient at the perivascular endfeet (reviewed in Liang et al., 2007). It is commonly though that it is this “ionic edema”, and the attendant increase in intracranial pressure that it creates, that is responsible for much of the morbidity and mortality associated with stroke. It has been suggested that AQP4 may serve as the primary conduit for the entry of water, and numerous studies have shown that interfering with AQP4 expression (Manley et al., 2000), localization (Amiry-Moghaddam et al., 2003, 2004), or function (Igarashi et al., 2010) can be an effective means of reducing the severity of the cerebral edema in the wake of an ischemic insult and improving animal survival and neurological outcome.

While current stroke therapies primarily focus on re-establishing blood flow to the affected portions of the brain, this may in itself lead to undesirable effects, including the generation of reactive oxygen species (ROS), which can inflict damage to membrane lipids, proteins and DNA (Aitken et al., 1993; Lopes et al., 1998; Levine et al., 2000; Mirzaei and Regnier, 2008). Additionally, it has been suggested that ROS may also trigger an increase in AQP4 expression (Rao et al., 2010), which would likely exacerbate cytotoxic edema. This is in agreement with the *in vivo* findings of certain groups, who observed increased AQP4 levels following medial cerebral artery occlusion (Taniguchi et al., 2000; Ribeiro Mde et al., 2006), while contradicting those of some others (Sato et al., 2000; Meng et al., 2004; Frydenlund et al., 2006; Friedman et al., 2009). The exact mechanisms that underlie this increase, however, currently remain obscure.

It is known that tyrosine 14 (Y14) of caveolin-1 (Cav1) undergoes phosphorylation by *Src* kinase under conditions of oxidative stress (Chen et al., 2005; Khan et al., 2006), and that this profoundly affects Cav1 function in the internalization and trafficking of a number of molecules, including a variety of receptor-tyrosine kinases and serpentine G-protein-coupled receptors, as well as epidermal growth factor receptor, insulin-like growth factor 1 receptor, and the lipid raft marker monosialotetrahexosylganglioside (GM1; Singh et al., 2003; Khan et al., 2006; Lajoie et al., 2007; Salani et al., 2010). Interestingly, we showed previously that AQP4 also codistributes with Cav1 in lipid raft enriched-fractions in the brain (Noël et al., 2009).

Given the above, we ask in the present study the question of whether Cav1 could play a role in regulating AQP4 targeting to the plasma membrane of astrocytes subjected to oxidative stress, using primary astrocyte cultures treated with hydrogen peroxide (H_2_O_2_) as a model. We first showed that H_2_O_2_ induces a significant increase in the expression of AQP4 protein levels and that this effect is inhibited by the antioxidant N-acetylcysteine (NAC). Second, using cell surface biotinylation, we demonstrated that H_2_O_2_ increases AQP4 plasma membrane expression and that this change is independent of *de novo* AQP4 synthesis. Finally, we found that the phosphorylation of Cav1 at Y14 is a regulator of H_2_O_2_-induced increase in AQP4 cell surface expression. To our knowledge, these findings are the first to show that Cav1 phosphorylation plays a key role in the regulation of AQP4 cell surface expression in oxidative stress, possibly by altering AQP4 internalization and trafficking, resulting in its redistribution to specific compartments of the cell.

## Experimental Procedures

### Antibodies and Reagents

The following antibodies were used in the present study: rabbit polyclonal anti-AQP4 targeting residues 249–323 of rat AQP4 (RRID:AB_2039734; catalog no. AQP-004, lot no. AQP004AN1302; Alomone Laboratories, Jerusalem, Israel), rabbit polyclonal anti-Cav1 raised against the N-terminus of the human sequence (RRID:AB_2072042; catalog no. sc-894, lot no. H0307; Santa Cruz Biotechnology, Santa Cruz, CA, USA), mouse monoclonal anti-Cav1-Y14 (RRID:AB_2244199 catalog no. 3251S, lot no. 2; Cell Signaling Technology, USA) and mouse monoclonal anti-β-actin (RRID:AB_476744; catalog no. A5441, lot no. 064K4790; Sigma-Aldrich, St.Louis, MO, USA). The antioxidant NAC was purchased from Sigma-Aldrich, while the protein biosynthesis inhibitor cycloheximide and the Src-family kinase inhibitor 4-Amino-5-(4-chlorophenyl)-7-(t-butyl)pyrazolo[3,4-d]pyrimidine (PP2), were purchased from Calbiochem (EMD Millipore, Billerica, MA, USA).

### Cell Culture

Primary astrocyte cultures were prepared from the hippocampi of 1-day-old Sprague-Dawley rat pups (RRID:RGD_734476; Charles River Laboratories International, Inc., Wilmington, MA, USA). Briefly, hippocampi dissected from the whole brain were cut into small pieces and dissociated via a 15-min incubation with 0.05% trypsin at 37°C (3.0 mg/ml; Gibco, Invitrogen, Burlington, ON, Canada). The resulting cell suspension was then transferred to 25 cm^2^ culture flasks containing Dulbecco’s modified Eagle’s medium (DMEM) supplemented with 10% fetal bovine serum, 1% penicillin-streptomycin and 1 mM L-glutamine (Gibco, Invitrogen, Burlington, ON, Canada) and allowed to proliferate over a 2-week period, during which the culture medium was changed every 3 days.

The MDA-435 cell lines expressing Cav1-mRFP, Cav1(Y14F)-mRFP and Cav1(Y14D)-mRFP (Joshi et al., 2012) (a generous gift from Drs. BJ and IRN, UBC, Vancouver, BC, Canada) were grown to confluence in media consisting of RPMI 1640 supplemented with 10% fetal bovine serum, 1% penicillin-streptomycin and 1% L-glutamine (Gibco, Invitrogen, Burlington, ON, Canada) over a period of 4 days on either on 12-well plates for assays involving the use of western blotting or 12-mm coverslips for immunofluorescence.

### Immunocytochemistry

Cells grown on poly-D-lysine-coated 12-mm glass coverslips were first washed with DPBS, and then fixed by immersion in 4% (w/v) formaldehyde in 0.1 M phosphate buffer for 8 min. They were then permeabilized and blocked for 1 h using a solution of 2% BSA and 0.3% Triton X-100 in PBS, and labeled successively with rabbit polyclonal anti-AQP4 (diluted 1/100 in the permeabilization solution described above) and Alexa Fluor 488 goat anti-rabbit IgG (diluted 1/700). Following that, the coverslips were mounted onto glass slides with Prolong Gold Antifade reagent containing DAPI, and labeling was visualized via confocal microscopy.

### Cell Surface Biotinylation

Biotinylation was performed using the method previously described (Tham and Moukhles, 2017). Briefly, confluent astrocyte and MDA-435 cultures were treated with H_2_O_2_ for 1 h, washed with cold DPBS (Gibco, Invitrogen, Burlington, ON, Canada), and then labeled for 30 min at 4°C using 0.5 mg/ml EZ-Link Sulfo-NHS-LC-Biotin (Pierce Biotechnology, Rockford, IL, USA). The reaction was quenched with 50 mM NH_4_Cl in DPBS for 10 min, following which the cells were extensively washed with DPBS, and then collected and pelleted. The cell pellets were lysed via a 1 h-long incubation in extraction buffer (25 mM Tris pH 7.4, 25 mM glycine, 150 mM NaCl, 5 mM EDTA, containing 1% Triton X-100 and complete protease inhibitor cocktail; Roche, Laval, QC, Canada). Nuclei and cellular debris were then removed from the suspension by centrifugation at 16,100 *g* for 10 min. A portion of this lysate, which contained the full complement of Triton-extractable proteins (representing the “input” fraction), was retained and denatured via a 1-min boiling treatment in reducing sample buffer (50 mM Tris pH 6.8, 2% SDS, 10% glycerol and bromophenol blue), while the remainder was incubated with streptavidin-conjugated agarose beads (Pierce Biotechnology, Rockford, IL, USA) to precipitate the biotinylated proteins. Part of the resulting supernatant, consisting primarily of non-biotinylated intracellular proteins (the “intracellular” fraction), was taken and denatured for later analysis. Biotinylated proteins bound to the streptavidin-agarose beads (the “cell surface” fraction) were then released and denatured using reducing sample buffer. The various fractions were then separated on the same sodium dodecyl sulfate-polyacrylamide gel electrophoresis (SDS-PAGE), and analyzed by immunoblotting (see below).

### Immunoblotting

Cells were first harvested and lysed in extraction buffer. The lysate was then centrifuged to remove cellular debris, following which the extracted proteins were denatured by boiling for 1 min in reducing sample buffer (see above). Samples were then separated using sodium dodecyl SDS-PAGE, and electrotransferred onto nitrocellulose membranes (Bio-Rad, Mississauga, ON, Canada), and probed with antibodies against AQP4 (diluted 1/1000), Cav1 (1/1000), Cav1-Y14 (1/1000) and β-actin (1/10,000). Bound antibodies were detected using horseradish peroxidase-conjugated goat anti-rabbit IgG or goat anti-mouse IgG (1/2000; Jackson ImmunoResearch Laboratories Inc., West Grove, PA, USA), and signals were visualized on Bioflex econo films (Interscience, Markham, ON, Canada) using chemiluminescence (ECL, GE Healthcare, Buckinghamshire, UK).

### Transfection

Transfection of MDA-435 wild type (WT) and mutant cells with the VSV-AQP4 plasmid (a generous gift from Jean Mérot, INSERM U533, Faculté de Médecine, Nantes, France) was performed using Lipofectamine 2000 (Invitrogen, Burlington, ON, Canada), with immunofluorescence assays taking place 48 h after.

To silence Cav1 expression, astrocytes were transfected in suspension before plating with 100 nM Cav1 siRNA (siGENOME and ON-TARGETplus SMARTpool siRNA reagents; Dharmacon Research, Inc., Thermo Fisher Scientific, Waltham, MA, USA) using Lipofectamine 2000 (Invitrogen, Burlington, ON, Canada). Scrambled Cav1 siRNA was used as a control (ON-TARGETplus siCONTROL nontargeting siRNA; Dharmacon Research, Inc., Thermo Fisher Scientific, Waltham, MA, USA). Forty-eight hours after plating, astrocytes were treated with 200 μM H_2_O_2_ for 1 h before being subjected to cell surface biotinylation.

### Quantitative and Statistical Analysis

Densitometric analysis was performed using Image Gauge 4.21 (Fujifilm, Tokyo, Japan). For cell surface biotinylation, we first normalized the signal intensity of cell surface AQP4 against that of AQP4 in the input fraction, then normalized the resultant ratio against those for the untreated controls. The overall significance of the data was determined via Student’s *t*-test. All statistical analysis was performed using GraphPad Prism 4.00 software (San Diego, CA, USA).

### Ethics Statement

Every procedure involving animals in this study was performed in accordance with the guidelines issued by the Canadian Council on Animal Care, and approved by the Animal Care Committee of the University of British Columbia (approval number A06-0319). Research animals were treated humanely, and euthanized efficiently with minimal handling in order to to minimize their distress and suffering.

## Results

### Hydrogen Peroxide Induces an Increase in AQP4 Protein Levels

**Figure 1:**
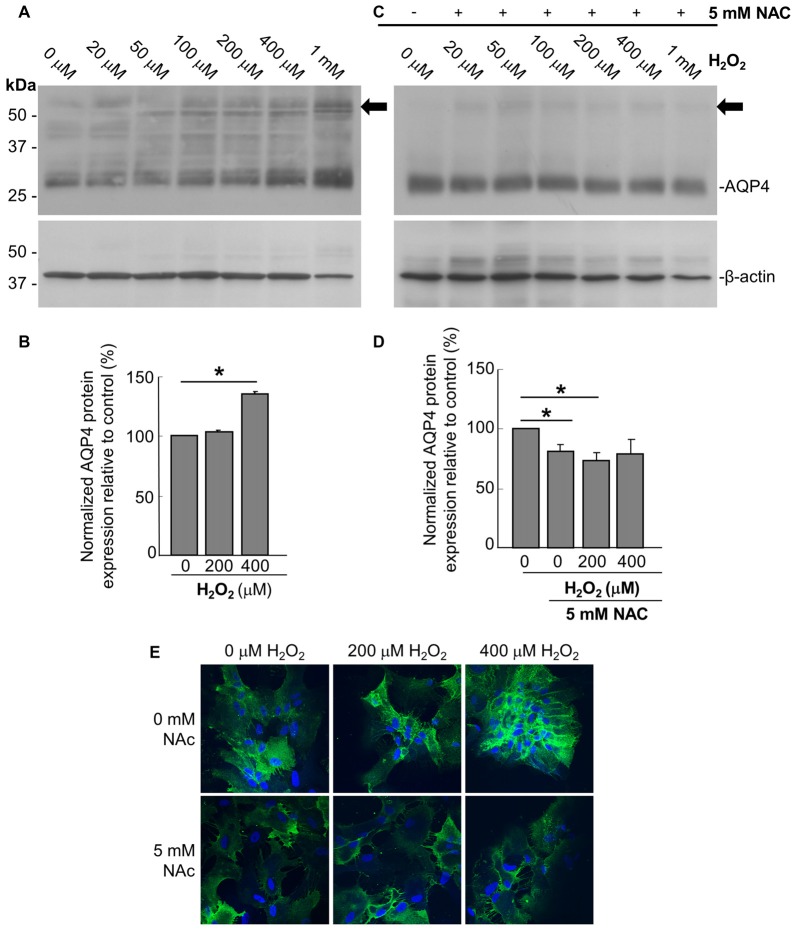
Hydrogen peroxide (H_2_O_2_) increases aquaporin 4 (AQP4) protein expression levels in astrocytes and this effect is reversed by the antioxidant, N-acetylcysteine (NAC). Representative immunoblot of AQP4 in primary astrocyte cultures treated for 1 h with increasing concentrations of H_2_O_2_, with β-actin shown alongside as a loading control **(A)**. Densitometric analysis illustrating the relative differences in AQP4 levels (normalized against that of β-actin) for select concentrations of H_2_O_2_ (**B**; averaged results of three independent experiments depicted). Immunoblot and quantification of AQP4 in astrocytes pre-incubated with the antioxidant NAC for 2 h, and then treated with H_2_O_2_ for 1 h (**C,D**; *n* = 3 independent experiments quantified in **D**). Arrows in immunoblots indicate the presence of multimers of AQP4. Statistically significant differences, as determined by the two-tailed Student’s *t*-test, are marked with symbols (*p < 0.05). Representative images of astrocytes incubated with H_2_O_2_ or preincubated with NAC for 2 h prior to H_2_O_2_, and immunolabeled for AQP4 **(E)**.

In order to assess the effect of oxidative stress on AQP4 expression, we analyzed primary astrocyte cultures treated for 1 h with increasing concentrations of H_2_O_2_ via immunoblotting, from which it was seen that H_2_O_2_ induces an upregulation of AQP4 protein levels (Figures 1A,B). This increase was abrogated when the cultures were pre-treated with 5 mM of the antioxidant and ROS inhibitor NAC for 2 h prior to the addition of H_2_O_2_ (Figures 1C,D). Interestingly, NAC reduced AQP4 levels by 19 ± 3% compared to controls not receiving NAC even in the absence of H_2_O_2_, indicating that AQP4 expression may be regulated by even the low levels of oxidative stress that exist endogenously under normal culture conditions. We repeated the above with two select concentrations of H_2_O_2_, this time visualizing differences in AQP4 using an immunocytochemical approach (Figure 1E, top panels). We saw that, in the absence of NAC, H_2_O_2_ increases the levels of AQP4-associated fluorescence, while this effect was abolished when the cells were preincubated with NAC (Figure 1E; bottom panels). Interestingly, a significant proportion of the increased AQP4 signal in the cells treated with H_2_O_2_ appeared to be associated with the plasma membrane.

### Hydrogen Peroxide Increases AQP4 Cell Surface Expression Independently of its *de novo* Synthesis

**Figure 2:**
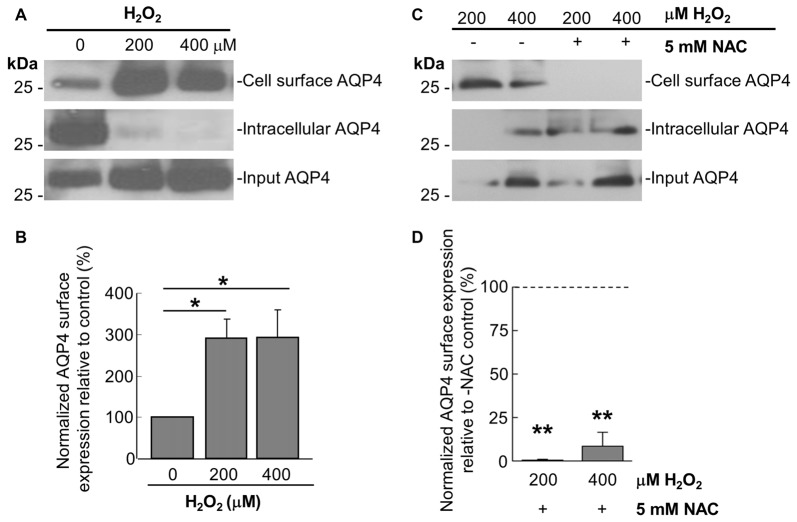
H_2_O_2_ increases AQP4 cell surface expression in a NAC-reversible manner. Immunoblots comparing cell-surface levels of AQP4 in astrocytes exposed to 200 and 400 μM H_2_O_2_ for 1 h **(A)** or preincubated with NAC for 2 h prior to H_2_O_2_ treatment **(C)**. Histograms depicting relative AQP4 cell-surface levels for the various conditions, normalized against the respective input levels for each condition (**B,D**; *n* = 3 independent experiments; *p < 0.05, **p < 0.01).

Prompted by the last finding, we thus performed cell-surface biotinylation studies on similarly-treated cells, through which it was determined that AQP4 expression at the membrane undergoes a near-three-fold increase in astrocytes treated for 1 h with 200 μM and 400 μM H_2_O_2_ (Figures 2A,B). This was accompanied by concomitant decreases in the intracellular levels of the channel in both cases (Figure 2A). Strikingly, preincubating the cells with NAC caused AQP4 cell-surface expression to decrease to barely-detectable levels compared to cells treated with H_2_O_2_ only (Figure 2C, quantified in 2D), indicating that oxidative stress may play a particularly central role in regulating AQP4 localization.

**Figure 3:**
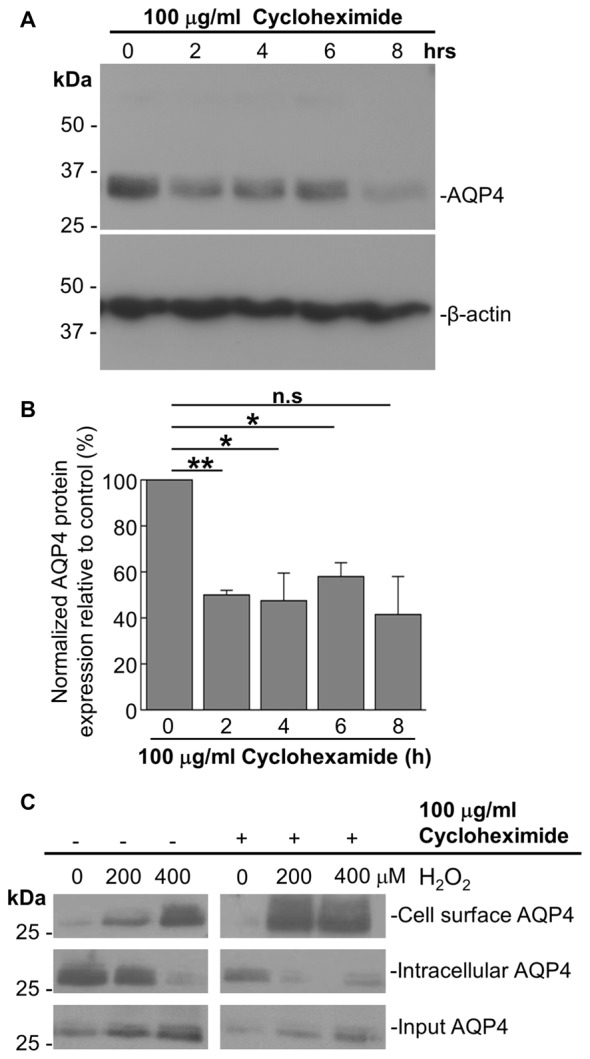
The H_2_O_2_-induced increase in AQP4 cell surface expression is independent of AQP4 synthesis. Total AQP4 levels in cells incubated with 100 μg/mL cycloheximide for the indicated lengths of time with β-actin as a loading control **(A)** and densitometric quantification of the same (**B**; *n* = 3 independent experiments; *p < 0.05, **p < 0.01). Cell-surface, intracellular and total (input) AQP4 in astrocytes treated for 1 h with only H_2_O_2_, or H_2_O_2_ following a 2-h pre-incubation with 100 μg/mL cycloheximide **(C)**.

As it was seen in the above that 200 μM H_2_O_2_ produces no detectable increase in the overall levels of AQP4 (Figure 1A), this raised the question of whether the above increase requires the expression of additional units of the channel. To address this, we repeated the above biotinylation assays on cells pre-incubated with 100 μg/mL cycloheximide for 2 h, a treatment that was empirically determined to result in the significant attenuation of AQP4 protein levels (Figures 3A,B), but saw that this had no significant impact on the increase in AQP4 cell-surface expression (Figure 3C). H_2_O_2_ therefore appears to enhance AQP4 membrane localization independently of *de novo* channel synthesis, which suggests that this increase is due rather to a redistribution of AQP4 from intracellular pools to the plasma membrane.

### Tyrosine Phosphorylation of Caveolin-1 Regulates H_2_O_2_-Induced Increase in AQP4 Cell Surface Expression

**Figure 4:**
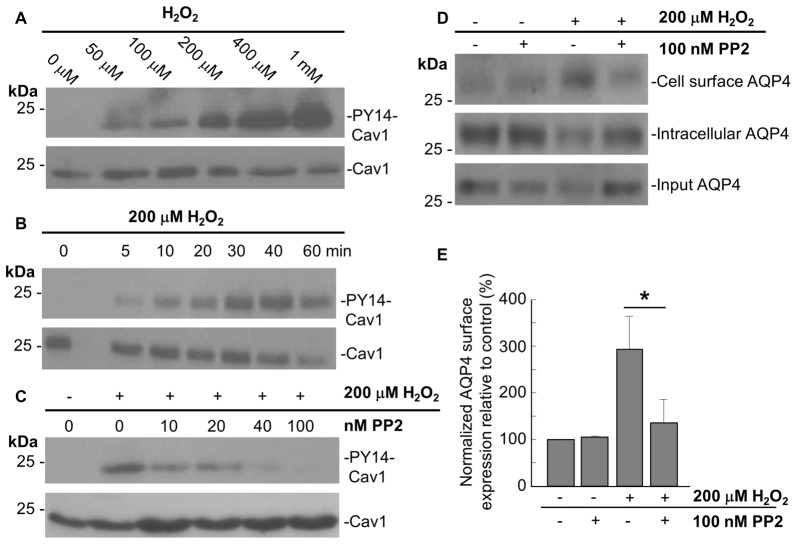
The Src kinase inhibitor 4-Amino-5-(4-chlorophenyl)-7-(t-butyl)pyrazolo[3,4-d]pyrimidine (PP2) suppresses the H_2_O_2_-induced increase in AQP4 expression at the cell surface. Immunoblots comparing the level of caveolin-1 (Cav1) Y14 phosphorylation in astrocytes exposed to various concentrations of H_2_O_2_ for 1 h **(A)**, or 200 μM H_2_O_2_ for increasing lengths of time **(B)**. Immunoblot depicting the effects of 1 h pre-incubation with increasing concentrations of PP2 prior to an hour-long treatment with 200 μM H_2_O_2_ on Cav1 Y14 phosphorylation **(C)**. Cell-surface, intracellular, and total AQP4 levels in control and PP2-pre-treated astrocytes in the presence and absence of a 1 h-long H_2_O_2_ treatment **(D)**, and a histogram depicting the relative, input-normalized cell-surface AQP4 amounts under these various conditions (**E**; *n* = 3 independent experiments; *p < 0.05).

In accordance with previous studies showing that acute oxidative stress induces the phosphorylation of tyrosine 14 of Cav1 in endothelial cells (Chen et al., 2005), we observed that Cav1-Y14 phosphorylation increased with H_2_O_2_ in a dose- and time-dependent manner in astrocytes (Figures 4A,B).

Cav1 is phosphorylated by the tyrosine-protein kinase Src in a wide variety of cell types (Glenney and Zokas, 1989; Li et al., 1996; Gottlieb-Abraham et al., 2013), and we found that the H_2_O_2_-induced phosphorylation of Cav1 decreases when astrocytes were pre-treated with the Src inhibitor PP2 for 1 h prior to H_2_O_2_ exposure. This was also dose-dependent, with the Y14 signal declining with increasing concentrations of PP2, until its complete abolishment at 100 nM of PP2 (Figure 4C).

To investigate if Cav1 phosphorylation might play a role in regulating AQP4 cell-surface expression under conditions of oxidative stress, we performed biotinylation assays on astrocytes that were first pre-treated with 100 nM PP2 for 1 h, and then exposed to H_2_O_2_ for 1 h, and found that the near-three-fold increase in cell-surface AQP4 normally elicited by H_2_O_2_ was much reduced (Figures 4D,E). Src-induced Cav1 phosphorylation therefore appears to be critical in mediating AQP4 increased cell surface expression in the presence of H_2_O_2_.

**Figure 5:**
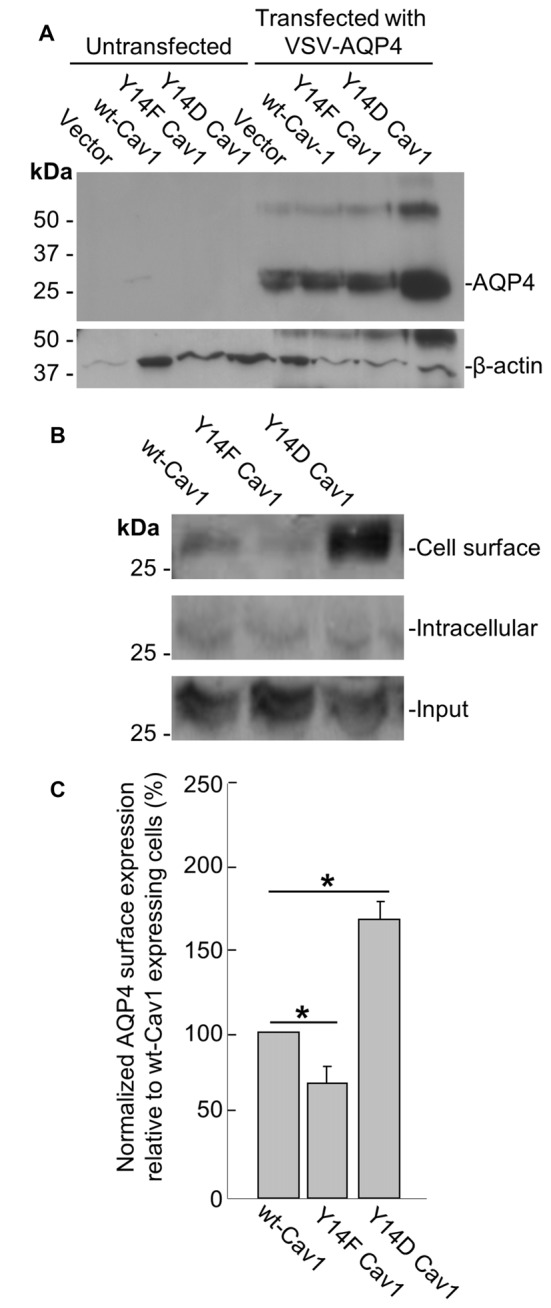
Cav1 Y14 phosphorylation enhances AQP4 expression at the cell surface in MDA-435 cells. Immunoblot demonstrating the expression of the VSV-AQP4 transgene in MDA-435 cell lines carrying an empty vector, or expressing wild-type (WT), Y14F (dominant-negative) and Y14D (phosphomimetic) variants of Cav1, with β-actin shown as a loading control **(A)**. Cell-surface, intracellular, and total AQP4 levels in MDA-435 cells expressing WT, Y14F and Y14D Cav1 **(B)**. Quantification of the above (**C**; *n* = 3 independent experiments; *p < 0.05).

To further address this issue, we transfected a plasmid containing VSV-tagged AQP4 into three MDA-435 cancer cell lines stably expressing WT Cav1, the dominant negative Y14F form, or one bearing the Y14D phosphomimetic mutation. After verifying via immunoblotting that AQP4 could be reliably expressed in all three lines (Figure 5A), we then used cell-surface biotinylation to determine how the cell-surface localization of AQP4 would be affected by Cav1 mutations. Consistent with our expectations, AQP4 membrane expression was decreased (by 31 ± 10%; *n* = 3) in cells expressing the dominant-negative Y14F Cav1 mutant compared to those expressing the WT form, whereas those expressing the Y14D phosphomimetic form showed a gain of 68 ± 10% (*n* = 3; Figures 5B,C).

**Figure 6:**
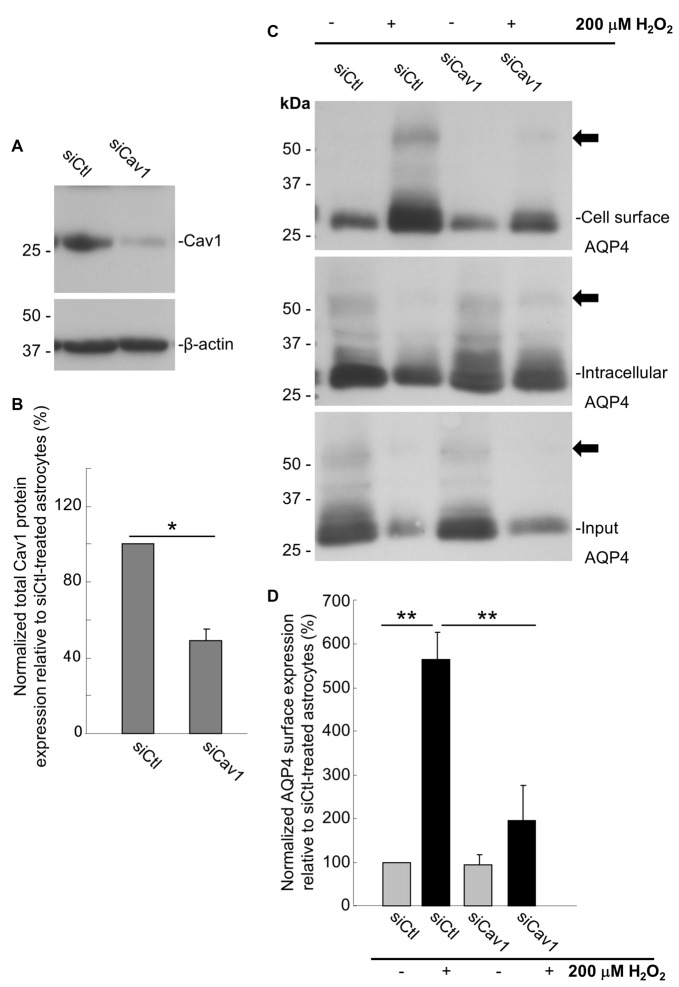
Loss of Cav1 inhibits the H_2_O_2_-induced increase in AQP4 cell-surface expression in astrocytes. Cav1 expression in control- (siCtl) and Cav1-siRNA (siCav1)-transfected astrocytes **(A)**. Quantification of the above (**B**; *n* = 3 independent experiments; *p < 0.05). Cell-surface, intracellular and total AQP4 levels in siCtl and siCav1-treated astrocytes in the absence and presence of a 1 h long H_2_O_2_ treatment **(C)**. A comparison of relative input-normalized average surface AQP4 levels for the above conditions (**D**; *n* = 4 independent experiments; **p < 0.01). Arrows in immunoblots indicate the presence of multimers of AQP4.

As a final evaluation of the role of Cav1, we transfected primary cultures of rat astrocytes with double-stranded siRNA targeting the rat Cav1 sequence (siCav1), which we found to be effective in decreasing Cav1 protein levels by 51 ± 6% (*n* = 3) on average (Figures 6A,B). While AQP4 cell-surface levels remained unaffected in the cells not treated with H_2_O_2_, Cav1 knock down resulted in a near-three-fold reduction (falling from 564 ± 45% to 194 ± 58%; *n* = 3; Figures 6C,D) of the normal H_2_O_2_-induced increase of AQP4 surface expression, conclusively demonstrating that Cav1 is required for AQP4 upregulation under oxidative stress.

## Discussion

AQP4 is highly-expressed in astrocytes, and may facilitate osmoregulation by mediating the bi-directional exchange of water between the brain and the bloodstream. It is also thought to be an important factor in the development of ischemia-induced brain edema. Given that ischemia results in an increase in ROS levels, we therefore chose in the present study to investigate if the latter and former might be linked, using H_2_O_2_-treated rat astrocyte primary cultures as a model system. We showed that H_2_O_2_ induces an increase in AQP4 protein levels, and in the cell-surface localization of the channel. We also demonstrated that Cav1 plays a key role in determining the cell-surface expression of AQP4 under conditions of oxidative stress, possibly through its regulation of channel internalization and trafficking.

### ROS Regulate the Cell-Surface Expression of AQP4 via Cav1 Phosphorylation

We showed here that the expression of AQP4 protein levels are upregulated in astrocyte cultures under oxidative stress conditions, and that this increase is abolished by the addition of the antioxidant and ROS scavenger NAC. It is not clear, in our experimental settings, how NAC accelerates the dissipation of H_2_O_2_. It could be via its role as a glutathione precursor by increasing glutathione synthesis (Townsend et al., 2003), or alternatively, by enhancing the transcription of oxidative stress response genes (Zaragoza et al., 2000). Either way, it is interesting to note that it had been demonstrated in a previous study that Edaravone, a free radical scavenger commonly prescribed for the treatment of ischemic stroke, inhibits AQP4 expression in the infarct area (Kikuchi et al., 2009), as does Resveratrol, a phytological polyphenol that may have antioxidant properties (Li et al., 2015). Similarly, vitamin E and catalase were reported to also be effective in abrogating a similar upregulation usually seen in astrocyte cultures damaged by fluid percussive injury (Rao et al., 2010).

As well as increasing AQP4 expression, we observed that H_2_O_2_ also caused the accumulation of the channel at the cell surface. Via the use of cycloheximide, we demonstrated that this increase can occur independently of an upregulation in *de novo* channel synthesis. This finding was consistent with that of a previous study, which showed that manganese-induced oxidative stress could elicit an increase in cell surface expression without affecting AQP4 protein levels (Rao et al., 2010).

The caveolins, a family of cholesterol-binding integral membrane proteins, are central components of both planar and caveolar lipid microdomains, or rafts. The membrane-shaping and scaffolding functions that these molecules provide are particularly essential for caveolar biogenesis (Drab et al., 2001; Walser et al., 2012), and their ability to modify raft cholesterol content (Roy et al., 1999), and modulate dynamin-mediated membrane scission (Henley et al., 1998; Le et al., 2002; Nabi and Le, 2003) also makes them key regulators of caveolar endocytosis. Numerous components involved in the signal transduction pathways associated with oxidative stress, such as Ras, Src-family kinases, and angiotensin II are contained within, or dependent on raft integrity as well (Matallanas et al., 2003; Cao et al., 2004; Adebiyi et al., 2014), and evidence suggests that they could also be the primary sites at which ROS are generated under certain circumstances (Gniadecki et al., 2002).

Oxidative stress can impinge upon caveolar transport processes. It was observed, for instance, that the phosphorylation of tyrosine 14 of Cav1 by Src kinase in pulmonary microvessel endothelial cells (PMVECs) exposed to oxidative stress leads to increased albumin uptake and transcytosis (Sun et al., 2009). The relationship between the phosphorylation state of Cav1 and endocytosis was explored further in a more recent study, in which it was demonstrated that the expression of the phosphomimetic Y14D mutant of Cav1 in the pancreatic beta cell line MIN6 caused accelerated insulin receptor uptake and degradation (Boothe et al., 2016). These findings are in agreement with Cav1 phosphorylation promoting the derepression of caveolin biosynthesis and the release of caveolae from the plasma membrane (Joshi et al., 2012; Zimnicka et al., 2016). While we confirm in the present study that Cav1 is similarly phosphorylated in H_2_O_2_-stimulated astrocytes, this induced AQP4 to be expressed at the cell surface in increased quantities, thus suggesting that phosphorylation may affect caveolin function quite differently in astrocytes than it does in PMVECs or MIN6 cells. Indeed, prior studies conducted by other groups have determined that, for unknown reasons, while Src-mediated tyrosine phosphorylation leads to Cav1 being translocated to detergent-insoluble membrane domains in PMVECs treated with bacterial lipopolysaccharide (Wang et al., 2015), the same modification has the opposite effect in H_2_O_2_-stimulated astrocytes, where it results in the exclusion of Cav1 from lipid rafts (Ito et al., 2015). The latter seemingly stands at odds with our previous finding that the application of the extracellular matrix protein laminin, a major component of the perivascular basal lamina interposed between the astrocytes and endothelial cells that comprise the BBB, to astrocyte cultures causes Cav1 to partially colocalize with clusters of β-dystroglycan, a receptor that is frequently co-expressed with AQP4, and simultaneously increases the cell-surface expression of the channel (Noël et al., 2009). These data are particularly striking in the present context, as laminin, like oxidative stress, similarly causes the accrual of AQP4 at the astrocytic plasma membrane (Tham et al., 2016). Although it is known that laminin-induced Cav1 phosphorylation occurs in melanoma cells, and is a central regulator of cell migration (Ortiz et al., 2016), it is unclear as to whether the same happens in astrocytes. Nevertheless, these results indicate that the pathways involved in the response to changes to extracellular matrix content or the level of oxidative stress may intersect and perhaps converge, with significant consequences on AQP4 function in astrocytes.

### AQP4 Levels May Be Regulated by Numerous Pathways during Oxidative Stress

Indeed, we have observed, both here (Figures 6C,D) and in the aforementioned study (Tham et al., 2016), that the attenuation of Cav1 expression by siRNA resulted in no discernable effects on cell-surface AQP4 levels in normoxic astrocytes and astrocytes not treated with laminin (Tham et al., 2016). It is possible that the remaining Cav1 following the partial knockdown was able to perform the function required in these states, but the likelihood that there exists a parallel pathway that is upregulated under such “baseline” conditions cannot be discounted either. Clathrin-driven endocytosis is a particularly strong candidate, especially as this pathway appears to be quite active in the portions of the astrocyte not in direct contact with the basal lamina, serving to maintain AQP4 amounts at relatively low levels in these domains (Tham et al., 2016).

The potential interaction between these two pathways is intriguing, particularly as it pertains to AQP4 regulation under oxidative stress. As it happens, c-Src, which is activated under these conditions, also positively regulates dynamin (Ahn et al., 1999), and thus one would expect clathrin-mediated endocytosis to also be accelerated in such instances. Yet, in our experiments, it was seen that H_2_O_2_ nonetheless induces an increase in surface AQP4 levels (Figure 2), indicating that the effects of H_2_O_2_ on Cav1 function is the overriding factor. This, taken in combination with the result that the effects of oxidative stress on AQP4 localization are abolished in siCav1 cells (Figure 6), suggests that Cav1 phosphorylation actively stabilizes AQP4 at the membrane. The manner in which these mechanisms act *in vivo* is likely to be less binary, and determined via the complex interplay of these pathways, with their relative contributions being decided by moment-to-moment changes in the local environment. This could account for why there is currently little consensus regarding whether AQP4 changes in expression levels is upregulated in animal models of stroke, as variations in the stroke model, the duration of reperfusion, and the methods used to determine AQP4 levels can all affect the outcome. For instance, it was observed in a recent study that, while AQP4 protein levels in the cortex are elevated by increasing levels of hypoxia, the effect is exacerbated by additional hypercapnia only in the animals that have undergone the more severe oxygen-deprivation treatments; paradoxically, CO_2_ caused a decrease in AQP4 expression in animals given air containing a near-normal amount of oxygen (Yang et al., 2016). Oxidative stress probably elicits a similarly graded response, the consequences of which may be therapeutically relevant.

While others had previously shown that oxidative stress can induce caveolin Y14 phosphorylation (Volonté et al., 2001; Sun et al., 2009), our study demonstrates that this phosphorylation can serve as an important regulator of cell-surface AQP4 levels in astrocyte cultures. However, it is uncertain whether the same is true *in vivo*. It is further unknown if the regulation of AQP4 cell-surface expression by Cav1 can significantly affect the cellular swelling associated with ischemia, especially given that this process may be dominated by osmotic gradients between oxidatively-stressed astrocytes and the extracellular space. A more thorough understanding of this pathway is therefore needed, and could reveal additional routes of AQP4 regulation *in vivo*.

## Author Contributions

CB designed and performed the majority of the experiments, collected and analyzed the bulk of the data and drafted the initial version of the manuscript. DKLT was involved in acquiring certain images and data analysis and revising the manuscript for submission. CP was responsible for compiling certain figures, data analysis and revising the manuscript. BJ created the cell lines carrying the phosphomimetic Cav1 mutants, provided suggestions on their use and reviewed the manuscript. IRN provided critical input on the design of the experiments involving the aforementioned cell lines, and on the interpretation of the results of these experiments. HM was responsible for the conception of this study, supervised the entirety of the project and the preparation of the manuscript.

## Conflict of Interest Statement

The authors declare that the research was conducted in the absence of any commercial or financial relationships that could be construed as a potential conflict of interest.
